# Temporal variability of spectro-temporal receptive fields in the anesthetized auditory cortex

**DOI:** 10.3389/fncom.2014.00165

**Published:** 2014-12-23

**Authors:** Arne F. Meyer, Jan-Philipp Diepenbrock, Frank W. Ohl, Jörn Anemüller

**Affiliations:** ^1^Medizinische Physik and Cluster of Excellence Hearing4all, Department of Medical Physics and Acoustics, Carl von Ossietzky UniversityOldenburg, Germany; ^2^Department of Systems Physiology of Learning, Leibniz Institute for NeurobiologyMagdeburg, Germany; ^3^Department of Neuroprosthetics, Institute of Biology, Otto-von-Guericke UniversityMagdeburg, Germany

**Keywords:** sensory coding, receptive field, inferior colliculus, auditory cortex, time-varying, zero-mean prior, generalized linear model

## Abstract

Temporal variability of neuronal response characteristics during sensory stimulation is a ubiquitous phenomenon that may reflect processes such as stimulus-driven adaptation, top-down modulation or spontaneous fluctuations. It poses a challenge to functional characterization methods such as the receptive field, since these often assume stationarity. We propose a novel method for estimation of sensory neurons' receptive fields that extends the classic static linear receptive field model to the time-varying case. Here, the long-term estimate of the static receptive field serves as the mean of a probabilistic prior distribution from which the short-term temporally localized receptive field may deviate stochastically with time-varying standard deviation. The derived corresponding generalized linear model permits robust characterization of temporal variability in receptive field structure also for highly non-Gaussian stimulus ensembles. We computed and analyzed short-term auditory spectro-temporal receptive field (STRF) estimates with characteristic temporal resolution 5–30 s based on model simulations and responses from in total 60 single-unit recordings in anesthetized Mongolian gerbil auditory midbrain and cortex. Stimulation was performed with short (100 ms) overlapping frequency-modulated tones. Results demonstrate identification of time-varying STRFs, with obtained predictive model likelihoods exceeding those from baseline static STRF estimation. Quantitative characterization of STRF variability reveals a higher degree thereof in auditory cortex compared to midbrain. Cluster analysis indicates that significant deviations from the long-term static STRF are brief, but reliably estimated. We hypothesize that the observed variability more likely reflects spontaneous or state-dependent internal fluctuations that interact with stimulus-induced processing, rather than experimental or stimulus design.

## 1. Introduction

Neurons in the auditory system must encode information about sensory stimuli exhibiting a wide range of statistical properties. Recent evidence suggests complex time-varying encoding mechanisms that facilitate this task, including adaptation to stimulus statistics in terms of regularity (Ulanovsky et al., 2003, 2004; Malmierca et al., 2009; Netser et al., 2011), (spectro-temporal) contrast (Escabi et al., 2003; Rabinowitz et al., 2011, 2012), sound level (Dean et al., 2005; Lesica and Grothe, 2008a), task-related plasticity (Fritz et al., 2003), learning-induced plasticity (Weinberger, 1993; Ohl and Scheich, 2005), or attentive modulation of the response (Ding and Simon, 2012; Mesgarani and Chang, 2012). These processes have been observed on time scales ranging from milliseconds to hours. An understanding of the underlying mechanisms is essential and requires techniques that allow robust characterization of neural coding, in particular on short time intervals.

The receptive field (RF) constitutes the classic, functional model relating sensory stimulus and evoked response of a neuron, for reviews see (Schwartz et al., 2006; Wu et al., 2006; Sharpee, 2013). However, the RF is primarily a function of bottom-up processing assuming the response to be unmodulated in the considered time interval, corresponding to estimation of a linear time-invariant system. Temporal changes in RF structure have been analyzed by producing RF estimates for different parts of an experiment (Fritz et al., 2003; Sharpee et al., 2006), or by recurrent linear filtering (Brown et al., 2001; Stanley, 2002). Techniques that exploit sparsity of RFs in a linear model have been developed to achieve distinct RF estimates with a comparably small amounts of data (Sahani and Linden, 2002; Machens et al., 2004; Park and Pillow, 2011). The resulting estimators allow the investigation of processing on smaller time scales than standard estimators. However, the representation of non-Gaussian stimuli in cortical areas mediated by non-linear transformations may often preclude application of these methods (David et al., 2004; Sharpee et al., 2006; Christianson et al., 2008; Meyer et al., 2014).

Here, we propose a novel approach to studying time-varying encoding mechanisms of sensory neurons. The focus is on neurons in the auditory system, which are commonly described by the spectro-temporal receptive field (STRF), the most probable spectro-temporal stimulus pattern that generated a measured spike response (Aertsen and Johannesma, 1981). Changes in STRF structure, as compared to it's static component, have been found to be small in studies of sound level encoding (Lesica and Grothe, 2008a), spectro-temporal contrast (Escabi et al., 2003; Rabinowitz et al., 2011), naturalistic stimulus processing in background noise (Lesica and Grothe, 2008b) and task-related plasticity in auditory cortex (Fritz et al., 2003). The time-varying “local” STRF, i.e., the STRF pattern best characterizing neuronal encoding within a short time-interval of experimental data, may therefore be represented as a superposition of it's long-term average and a time-dependent deviation. The former, denoted here as “static” STRF, is computed from the whole ensemble of available data, using methods that allow robust RF estimation for stimulus ensembles with second- and even higher-order correlations across stimulus dimensions (Paninski, 2004; Sharpee et al., 2004; Truccolo et al., 2005; David et al., 2007; Calabrese et al., 2011; Fitzgerald et al., 2011; Meyer et al., 2014). The superposition Ansatz, thus, reduces the problem of identifying the “local” STRF to the problem of identifying temporally-localized deviations from the long-term “static” STRF and the formulation of a suitable statistical model.

A common technique in RF estimation is to bias RF estimates toward solutions that are more probable a priori (Sahani and Linden, 2002; David et al., 2007; Park and Pillow, 2011). Sparsity is a special form of prior that biases RF parameters toward zero, e.g., by assuming a zero-mean Gaussian distribution of RF parameters (Machens et al., 2004). We adopt this technique and formulate a prior that biases local STRF estimates toward an a priori more probable STRF, specifically the static STRF estimate obtained from a long stimulus sequence as described above. For a given neuron, the static STRF prior is likely more informative than, e.g., a sparseness prior since RFs show comparably small variation between different stimulus conditions (e.g., Fritz et al., 2003; Sharpee et al., 2006). This prior is denoted “adaptive prior” because it allows the static RF estimate to adapt to temporally localized structures in the data. The formulation in terms of a prior allows us to incorporate the approach into linear as well as non-linear models, e.g., the generalized linear model [GLM, Nelder and Wedderburn (1972)], which allows RF estimation under more general conditions than linear models (Paninski, 2004; Truccolo et al., 2005; Pillow et al., 2008).

We derive closed-form solutions for the linear case and show that when the stimulus ensemble contains strong second-order correlations, a combination of zero-mean and adaptive prior, denote “mixed prior,” may improve local RF estimates significantly. Further, we combine adaptive and mixed priors with a GLM. The resulting estimator allows conservative but robust characterization of time-varying neural processing, even for highly non-Gaussian stimuli like human speech that may lead to highly biased STRF estimates for linear estimators. We also apply the proposed approach to recordings from auditory midbrain and auditory cortex in anesthetized Mongolian gerbils. We address the following questions. First, do neurons at different levels in the auditory system perform time-invariant integration of stimulus features when probed with a complex, dynamic stimulus ensemble? Second, if so, does a time-varying model of sensory feature integration provide a better description of neural processes than a static model? Finally, if response characteristics of neurons are time-dependent, do changes occur deterministically or largely at random? Finding answers to these questions may provide more insight into time-varying encoding of biologically relevant information.

## 2. Materials and methods

### 2.1. Neural coding model

Characterizing a neuron's response to sensory stimuli involves presenting stimuli from a fluctuating stimulus ensemble and recording the evoked response. To simplify matters, we assume that the response has already been discretized in time bins of duration Δ, and *r_i_* ∈ {0, 1} indicates whether or not a spike has been observed in the *i*^th^ time bin. The history of stimulus features preceding the response in the *i*^th^ time bin, e.g., intensity values of an image patch or the spectro-temporal density of a sound, is summarized in the vector **s**_*i*_.

In a simplified model, we may assume that a neuron integrates stimulus features in terms of a linear filter **k**, the receptive field (RF), and the filtered stimulus, *z_i_* ≡ **s**^T^_*i*_**k**, is transformed into a neural response using a static, memoryless non-linearity *f*. Such a cascade is known as linear-nonlinear (NL) model (Hunter and Korenberg, 1986; Chichilnisky, 2001), and the probability of observing a spike in time bin *i* is given by

(1) $$p(\text{spike}|s_{i})\propto f\left(z_{i}\right).$$

**Figure 1:**
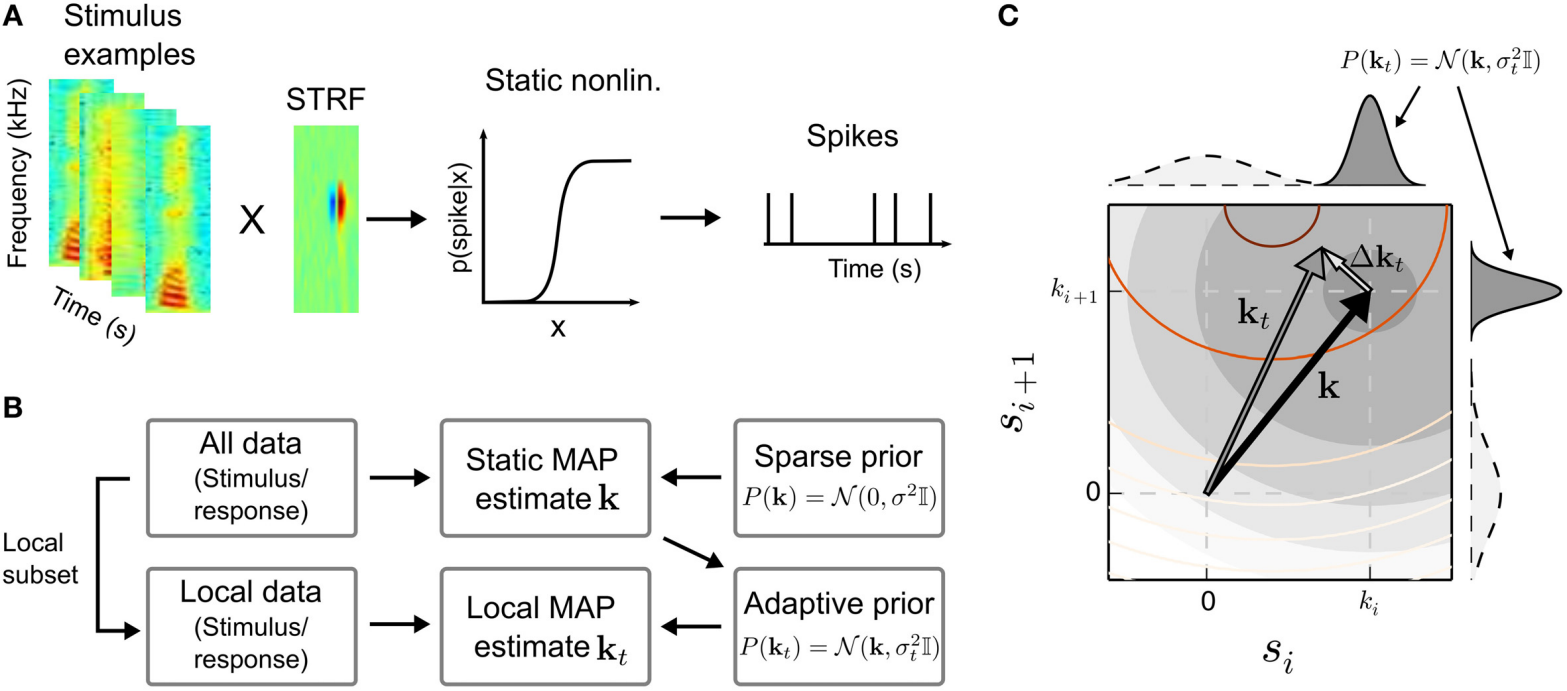
**Estimation of time-varying spectro-temporal receptive field (STRF) parameters in the linear-nonlinear model. (A)** In the linear-nonlinear model, it is assumed that stimulus examples, represented here by spectro-temporal patches sampled from a speech spectrogram, are filtered by a linear filter **k**, the STRF. The output of the linear stage is transformed by a static non-linearity into a spike rate. In the generative model, a binary spike response is obtained by a subsequent inhomogeneous Poisson process. **(B)** A typical approach to infer the parameters of the model is the maximum a posteriori (MAP) estimate. The MAP allows to incorporate prior information, e.g., a prior that enforce sparseness by biasing STRF parameters toward zero by assuming a zero-mean Gaussian distribution of STRF parameters **k**. In the scenario of time-dependent neural processing, the MAP estimate obtained using all data, which constitutes the most probable static solution, may in turn be used as informative prior for a “local” (or time-dependent) MAP estimate **k**_*t*_. Local evidence will result in deviations from the static STRF. **(C)** Illustration of the relation between static STRF **k** (black arrow) and local STRF **k**_*t*_ (gray arrow) in two stimulus dimensions *s_i_* and *s*_*i* + 1_. In case the likelihood (reddish contour lines) systematically deviates from the static prior (filled gray contours and gray marginal distributions), the maximum of the MAP estimate will be shifted by Δ**k**_*t*_ resulting in a time-varying local STRF. The relatively flat marginal distributions of the sparse (zero-mean) Gaussian prior (dashed lines) indicate that the sparse solution would be dominated by the likelihood, which may be difficult to estimate for small sample sizes. For visualization purposes, the contour lines show likelihood and static prior on a logarithmic scale.

A binary spiking response may be obtained by an inhomogeneous Poisson process that is modulated by *p*(spike|**s**) (Chichilnisky, 2001). Figure 1A illustrates the cascade model for the STRF.

Neural processing, in particular in cortical areas, has been found to be adaptive, and a static RF may not be sufficient to account for neural responses (Brown et al., 2001; Stanley, 2002; Escabi et al., 2003; Sharpee et al., 2006; Rabinowitz et al., 2011; Mease et al., 2013). A common approach is to estimate the parameters of the model Equation (1) for different parts of the data, assuming a short-term time-invariance approximation of the system (Fritz et al., 2003; Sharpee et al., 2006). Low temporal resolution is associated with the approach since a sufficient amount of data is needed to estimate the RF, thus adaptation that occurs on a faster timescale may not be identified reliably. Here, we propose an alternative approach that allows the linear RF in Equation (1) to adapt to temporally localized structures in the data according to the additive model

(2) $$k_{t}=k+\Delta k_{t},$$

where a temporally localized deviation Δ**k**_*t*_ is superimposed on the long-term static RF estimate **k**. We note that *t* indicates a time interval (in contrast to a single observation) and **k**_*t*_ is the “local receptive field” (local RF) that is assumed static during this (brief) time interval. Thus, each local RF **k**_*t*_ characterizes the response to a contiguous subset of the stimulus-response ensemble denoted by {*r_i_*, **s**_*i*_: *i* ∈ *N_t_*}.

### 2.2. Prior-based learning of time-varying receptive field parameters

Let us assume that we have estimated a static RF **k** for a neuron. Even in case neural processing is time-varying, **k** constitutes the most probable stimulus pattern to which the neuron is sensitive across the whole stimulus-response sequence. Thus, **k** may be considered the most likely a priori description of the neuron for every part of the data. However, local evidence in the data may result in systematic deviations from the static RF. If we assume that small fluctuations are more probable than large fluctuations, the relation between static RF and local RFs may be formulated as prior over local RF parameters. This is outlined in Figure 1B. Having no prior reason to favor either positive or negative deviations, we use an isotropic multivariate Gaussian prior centered around the static RF,

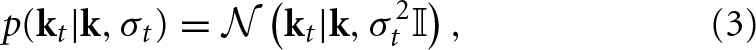



with standard deviation σ_*t*_ and identity matrix 𝕀. The time index indicates that σ_*t*_ may vary from part to part.

Figure 1C illustrates the relation between local deviation Δ**k**_*t*_ from the static RF and the prior on local RF parameters. If the static RF **k** is in agreement with the local data given by the likelihood, σ_*t*_ will be small and **k**_*t*_ will be very close to **k**. Otherwise, the prior distribution will be rather wide, allowing stronger deviations from **k**. The estimate may become less reliable due to the higher dispersion of the prior distribution. We will demonstrate that allowing local RF parameters to be either adaptive or zero-mean may increase robustness in such situations.

The probabilistic formulation in terms of a prior allows to apply the principle to a wide range of models that can be formulated in the maximum a posteriori (MAP) framework. This includes linear as well as non-linear models such as the GLM. For a general form of the likelihood function, *p*(**r**_*t*_|**S**_*t*_, **k**_*t*_), where the matrix **S**_*t*_ contains all stimulus examples in part *t* and **r**_*t*_ the corresponding response values, the MAP estimate can be written as

(4) $${\hat{k}}_{t}|k,\sigma_{t}=\;\underset{k_{t}}{\arg\;\max}\;p(r_{t}|S_{t},k_{t})p(k_{t}|k,\sigma_{t}).$$

where *p*(**k**_*t*_|**k**, σ_*t*_) is the prior on **k**_*t*_ and the MAP estimate is the mode of the posterior. Thus, we use a highly informative prior on the local RF to obtain a robust estimate of the maximum of the posterior. As data size increases, the likelihood overwhelms the prior and converges, similarly to the static estimator, toward the maximum likelihood estimate.

### 2.3. The linear-Gaussian case

If the response of a neuron may be described by a linear function, the stimulus-response relation can be written as

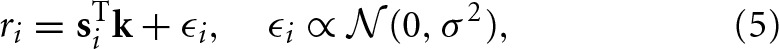



where ϵ_*i*_ is a zero-mean Gaussian white noise (GWN) sample with standard deviation σ. Here, *r* is assumed to be a continuous variable, e.g., the average number of spikes for several stimulus repetitions. For a complete measurement with *N* stimulus-response pairs, the likelihood that the response **r** = [*r*_1_, *r*_2_, *r*_3_, …, *r_N_*] is generated by **S** = [**s**_1_, **s**_2_, **s**_3_, …, **s**_*N*_]^T^ and the RF **k** is given by

(6) $$p(r|S,k)=\frac{1}{\sqrt{2\pi }\sigma }\exp\;\left(-\frac{1}{2\sigma^{2}}{\left(r-Sk\right)}^{\text{T}}\left(r-Sk\right)\right)$$

with maximum likelihood (ML) estimate

(7) $$\hat{k}={\left(S^{\text{T}}S\right)}^{-1}\left(S^{\text{T}}r\right).$$

The above estimator allows RF estimation from Gaussian stimulus ensembles. Correlations across different stimulus dimensions are “rotated out” using the inverse of the covariance matrix (Paninski, 2003).

#### 2.3.1. The “zero-mean” Gaussian prior

Sparseness is a distinct property of RFs and exploiting sparseness may improve performance of an estimator significantly (Sahani and Linden, 2002; David et al., 2007; Calabrese et al., 2011; Park and Pillow, 2011). A simple form of sparseness assumes an isotropic multivariate Gaussian distribution centered at zero with standard deviation σ_*a*_, *p*(**k**|σ_*a*_) = 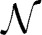 (**k**|**0**, σ^2^_*a*_ 𝕀). The MAP estimate is given by

(8) $${\hat{k}}_{\text{zero–mean}}|\alpha ={\left(S^{\text{T}}S+\alpha 𝕀\right)}^{-1}\left(S^{\text{T}}r\right)$$

with $\alpha \equiv \frac{\sigma^{2}}{\sigma_{\alpha }^{2}}$ (Hoerl, 1962). The problem in Equation (8) is also known as ridge regression, regularized linear regression, and penalized least squares (Hastie et al., 2001). Regularization is controlled by the hyper parameter α. For α → ∞, Equation (8) effectively computes the cross-correlation between stimulus and response, the spike-triggered average [STA, deBoer and Kuyper (1968)]. The “naive” ML estimator in Equation (7) arises for α = 0. Often, α is found by cross-validation (Machens et al., 2004).

#### 2.3.2. The “adaptive” Gaussian prior

The time-varying RF model in Equation (2) assumes that the time-dependent RF **k**_*t*_ can be described by time-dependent deviations from the static RF **k**. In a probabilistic model, these deviations from the static RF can be expressed in the form of a prior that uses the static RF as most probable solution. Thus, instead of a Gaussian prior distribution with zero-mean, we may use a Gaussian centered around the static RF **k** and use this as prior distribution in the MAP estimate in Equation (4).

In the linear-Gaussian model, the MAP estimate under the adaptive prior is given by

(9) $${\hat{k}}_{t}|k,\beta_{t}={\left(S_{t}^{\text{T}}S_{t}+\beta_{t}𝕀\right)}^{-1}\left(S_{t}^{\text{T}}r_{t}+\beta_{t}k\right)$$

with $\beta_{t}\equiv \frac{\sigma^{2}}{\sigma_{\beta_{t}}^{2}}$, and σ^2^_β_*t*__ is the variance of the Gaussian prior in Equation (3). The estimator includes the sum of the STA and a term that enforces adaptation to the static solution. The balance between both terms, which determines the amount of adaptation to **k**, is controlled by the hyperparameter β_*t*_. Again, β_*t*_ may be found by cross-validation on the training data.

#### 2.3.3. Mixing both priors: the “mixed” Gaussian prior

In many situations, it is necessary to control the amount of regularization and adaptation independently. In particular in case the employed stimulus ensembles contains strong second-order correlations across stimulus dimensions. However, when the adaptive prior distribution has a high dispersion, i.e., a large standard deviation, the estimator in Equation (9) will approach the non-regularized “naive” ML estimator (Equation 7) instead of the regularized ridge solution (Equation 8). In many situations, the regularized solution provides a more robust characterization of response properties than the ML estimator (Sahani and Linden, 2002; Sharpee et al., 2006; Park and Pillow, 2011).

Assuming that both priors are independent, the “mixed” prior, given by the product of zero-mean and adaptive prior,

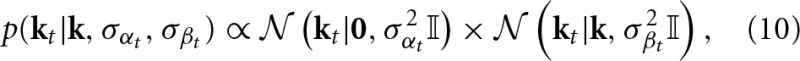



may improve local RF estimates in such situations. Noting that the product of two Gaussian distributions is also Gaussian, the resulting estimate for **k**_*t*_ can be written as (see Appendix for details)

(11) $${\hat{k}}_{t}|k,\lambda_{\alpha_{t}},\lambda_{\beta_{t}}={\left(S_{t}^{\text{T}}S_{t}+(\lambda_{\alpha_{t}}+\lambda_{\beta_{t}})𝕀\right)}^{-1}\left(S_{t}^{\text{T}}r_{t}+\lambda_{\beta_{t}}k\right),$$

where $\lambda_{\alpha_{t}}\propto \frac{\sigma^{2}}{\sigma_{\alpha_{t}}^{2}}$ and $\lambda_{\beta_{t}}\propto \frac{\sigma^{2}}{\sigma_{\beta_{t}}^{2}}$. Thus, the MAP estimate depends on two hyper parameters, and the estimator may vary between zero-mean (λ_β_*t*__ = 0) and adaptive solution (λ_α_*t*__ = 0).

#### 2.3.4. Simulated example

To demonstrate the performance of the MAP estimators with the different priors, we simulated data in the linear-Gaussian model (cf. Equation 5). The linear filter of the model cell represents a temporal onset RF with symmetric positive and negative amplitudes. We probed the cell with a short Gaussian noise sequence (*N* = 1000 samples). The correlation length of the Gaussian noise what about the length of the temporal filter. Spikes were created from the RF-filtered stimulus by an inhomogeneous Poisson process. Suppose, we have also probed the neuron with a longer sequence and the hypothetical static RF estimate systematically deviates from the RF used to generate the data for the short sequence. In such a case, the “adaptive” and “mixed” priors may use the static RF estimate for the long sequence as the prior during inference of RF parameters for the short sequence.

**Figure 2:**
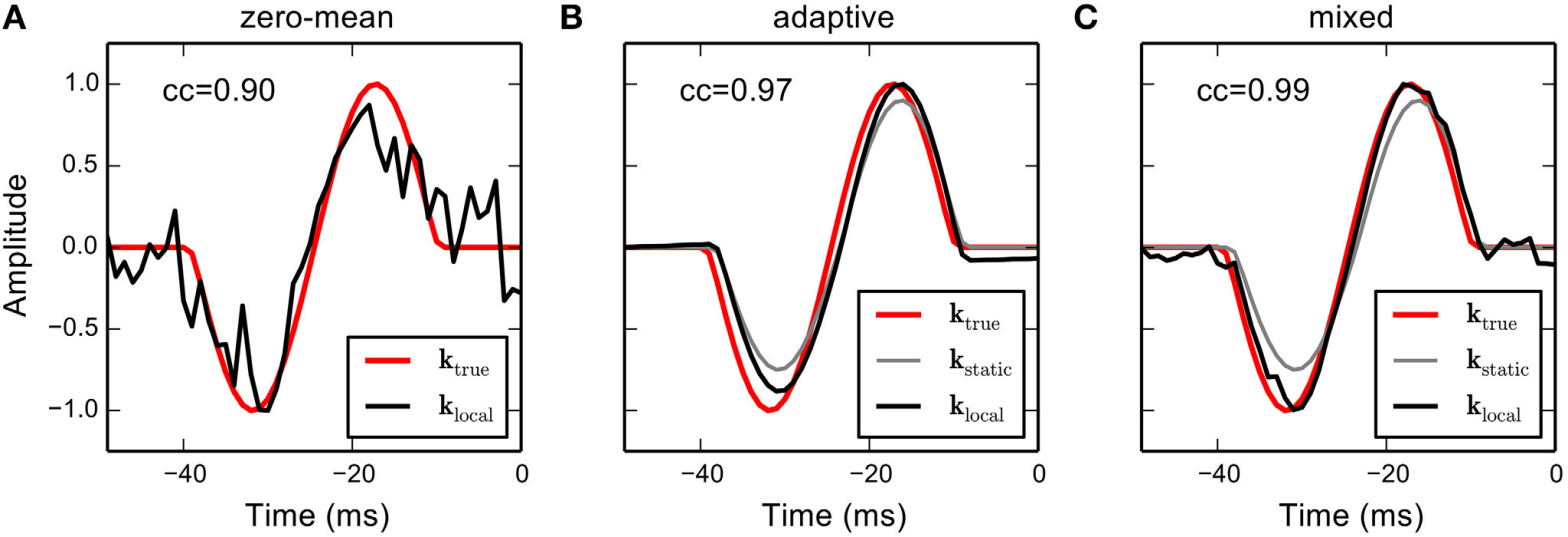
**RF estimation from simulated responses using the different priors in a linear-Gaussian model**. A strongly correlated Gaussian noise sequence (*N* = 1000 samples) was filtered with a temporal linear RF (red solid line). Spikes were generated by from the filtered stimulus by an inhomogeneous Poisson process resulting in 124 spikes. **(A)** The RF estimate obtained using a sparse (zero-mean) Gaussian prior on RF parameters. The estimate is very noisy due to the small number of observations. **(B)** Suppose we have estimated a static RF for the same cell, and the RF shows a different scaling and a slightly different latency (gray line). The adaptive prior biases RF coefficients toward the static RF. The resulting RF estimate is very smooth and partially adapts to the rescaled amplitudes but does not account for temporal shift and asymmetric amplitude scaling. **(C)** The mixed prior uses an additional hyper parameter that allows to control the trade-off between zero-mean and adaptive priors. The resulting estimator allows to account for the temporal shift at the expense of a slight increase in noise. Numbers in the upper left corner indicate correlations between estimated and true RF.

Figure 2 shows RF estimates based on the different priors. The hyperparameters in the adaptive and mixed priors were found using cross-validation. The estimate obtained using the zero-mean Gaussian prior approximately recovers the true RF but is very fuzzy due to the small number of samples in the short sequence. The RF produced by the adaptive prior is very smooth and reveals strong adaptation to the static RF, which exhibits asymmetric amplitude scaling and a slight shift in latency. The mixed prior combines the benefits of both estimates, resulting in an estimate that closely resembles the structure of the true RF. Thus, incorporation of prior knowledge, even in case the true local RF systematically deviates from the static RF, may significantly increase the quality of the RF estimate obtained from small data samples.

### 2.4. The non-Gaussian case: extending the generalized linear model (GLM)

The GLM framework allows RF estimation under more general conditions than linear estimators (Nelder and Wedderburn, 1972). In particular, the GLM allows for different non-linearities, incorporation of spike-interactions in the form of a post-spike filter (Paninski, 2004; Truccolo et al., 2005; Calabrese et al., 2011), and correlations between different neurons (Pillow et al., 2008). Under a GLM, the likelihood for the response *r* is chosen from an exponential family of distributions, and the expected output is given by

(12) 𝔼[r|s]=f(z),

where *f* is an invertible non-linearity and *z* = **s**^T^**k** + *b* relates the input stimulus **s** to the input of the non-linearity through the RF **k** and an optional offset term *b*.

Here, we adopt the concept of the adaptive prior to a special case of GLM, the linear-nonlinear Bernoulli model (Truccolo et al., 2005; Gerwinn et al., 2010). In the Bernoulli GLM, it is assumed that the output is distributed according to a Bernoulli distribution with likelihood

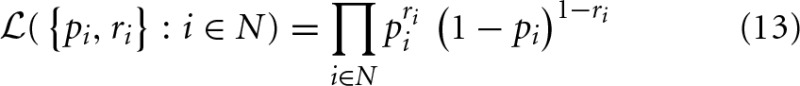



with *p_i_* ≡ *p*(*x_i_*), and *r_i_* ∈ {0, 1} denotes the presence (*r_i_* = 1) or absence (*r_i_* = 0) of a spike, respectively. The canonical non-linearity corresponding to the Bernoulli assumption is given by the logistic function

(14) $$f(z)=\frac{1}{1+{\text{e}}^{-z}}.$$

The log-likelihood for a local RF estimate at time *t* is given by

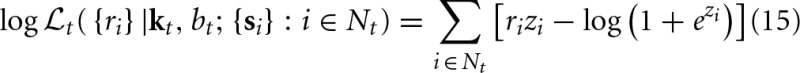



with *z_i_* ≡ **s**_*i*_^T^**k**_*t*_ + *b_t_*. In an analogy to the linear case, we may extend the GLM by a zero-mean, adaptive or mixed prior. For the mixed prior, the local MAP estimate is given by

(16) $$\begin{array}{l} k_{t}^{*}|k,\alpha ,\beta =\underset{k_{t},b_{t}}{\arg\;\max}\sum_{i\;\in \;N_{t}}\left[r_{i}z_{i}-\log\left(1+e^{z_{i}}\right)\right]\\ \;-\frac{\alpha }{2}k_{t}^{\text{T}}k_{t}-\frac{\beta }{2}{\left(k_{t}-k\right)}^{T}\left(k_{t}-k\right) \end{array}$$

with regularization hyperparameters α and β. The Gaussian prior is not the conjugate prior to the Bernoulli distribution. Consequently, there is no closed-form solution to Equation (16). However, the penalized log-likelihood function is convex, and the parameters can be found using gradient descent. We used a trust region Newton conjugate gradient algorithm (Lin et al., 2008) to find a solution for a set of hyper parameters α and β. The values for α and β that maximize the Bernoulli log-likelihood in a 5-fold cross-validation (CV) scheme were used to estimate the final model parameters.

### 2.5. Electro-physiological recordings

#### 2.5.1. Ethics statement

All experiments were conducted in accordance with the international National Institutes of Health Guidelines for Animals in Research and with ethical standards for the care and use of animals in research defined by the German Law for the protection of experimental animals. Experiments were approved by the local ethics committee in the state Saxony-Anhalt, Germany.

#### 2.5.2. Physiology experiment

Single-unit recordings were made in inferior colliculus (IC) and in the primary auditory cortex (A1) in ketamine-anesthetized Mongolian gerbils. Recordings were performed in an acoustically and electrically shielded recording chamber. Sounds were delivered by an amplifier to a calibrated Canton Plus XS.2 speaker. Single-unit recordings were made using a single tungsten electrode (3–4 MΩ) and digitized using a multichannel recording system (Multichannel Acquisition Processor; Plexon). Single spiking units were verified off-line (SpikeSorter; Plexon). Only units with stable waveform throughout the recording and spike count rate of at least two spikes per second were included in the analysis. This resulted in 30 units for IC and A1, respectively. For a detailed description of experimental procedures see Happel et al. (2010) and Meyer et al. (2014).

Stimuli consisted of frequency-modulated (FM) tone complexes (Meyer et al., 2013, 2014). The FM tones were arranged in consecutive 100 ms blocks, and each block contained four FM tones with randomly chosen starting and ending frequencies. At the beginning and the ending of each block a half cosine ramp of 5 ms was applied to prevent onset and offset artifacts. The frequencies were drawn from the interval 0.5 kHz to 16 kHz such that their distribution is flat on a logarithmic scale. Thus, the average spectrum is approximately constant for the auditory filter bank used for analysis (see below). The sampling frequency of the acoustic waveforms was 44.1 kHz. Each stimulus had a duration of 10 s and subsequent stimuli were interleaved with 100–1000 ms of silence. The total length of the stimulus sequence was between 300 s and 500 s for the IC recordings and between 180 s and 1000 s for the A1 recordings.

#### 2.5.3. Data preprocessing

As a first step of the data analysis, all stimuli were transformed into the time-frequency domain using a gammatone filter bank with octave-like frequency resolution (Hohmann, 2002). The magnitude of the complex-valued filter bank output was resampled to *f_s_* = 400 Hz for IC data and *f_s_* = 250 Hz for A1 data, yielding a spectrogram representation of the acoustic stimulus. The resulting bin sizes used to quantize spike times were Δ*t* = 2.5 ms for IC data and Δ*t* = 4 ms for A1 data. To account for dynamic properties of the cochlea, the resampled filter bank outputs were compressed using a static logarithmic function (Gill et al., 2006).

Stimulus examples were created from the spectrogram by recasting spectro-temporal patches preceding the response in a specific time window as vectors **s**. Thus, the vector **s**_*i*_ contained the part of the spectrogram that preceded the time step *i*Δ*t*, *i* = 1, 2, 3, …, up to *T*_win_. We used *T*_win_ = 50 ms and *T*_win_ = 100 ms for IC and A1 data, respectively. The spike trains were aligned to the time bins of the filtered stimuli, i.e., a spike at time *t_s_* was assigned to the *i*^th^ time bin [(*i* − 1)Δ*t*, *i*Δ*t*) if (*i* − 1) Δ*t* ≤ *t_s_* < *i*Δ*t*. We never observed more than one spike per time bin for both IC and A1 recordings. Thus, the response can be considered essentially binary, and the Bernoulli GLM is an adequate candidate for modeling the data.

#### 2.5.4. Correlation between STRFs

We quantified similarity of STRFs in terms of the correlation between the STRF estimates (Sharpee et al., 2004; Fitzgerald et al., 2011). For two *D*-dimensional vectors **u** and **v**, the correlation is given by

(17) $$\text{cc}=\frac{u^{\text{T}}v}{\|u{\|}_{2}\|v{\|}_{2}}$$

where $\|u{\|}_{2}={\left(\sum_{i}^{D}u_{i}^{2}\right)}^{1/2}$ denotes the *L*_2_-norm. The quantity cc describes the correlation between **u** and **v**, with cc = 1 denoting perfect correlation and cc = 0 denoting uncorrelated (orthogonal) vectors. For simulated data, **u** and **v** may represent true and estimated RF, respectively. On neural recordings, we used this quantity to describe similarities between static and local STRF estimates.

#### 2.5.5. Evaluation of prediction performance

We quantified prediction performance on neural data by keeping back a random set of test data and performing predictions on these data. The remaining data were used as training set. Predictions were done using static STRFs or time-varying STRFs (based on local STRF estimates). We used a cross-validation scheme similar to repeated random subsampling. For every data set, a random sample of 10% of the data was kept back as validation set and a static STRF was estimated using the 90% training data. We strove to have the same fraction of spikes examples in both the training and the validation set. Local STRFs were estimated by subdividing the whole data set into non-overlapping parts (20 s), removing the validation examples, and learning STRF parameters using the estimated static STRF in the mixed prior. Thus, the validation set has not been used for learning both static and local STRFs. This procedure has been repeated for different part offsets *t*_0_, i.e., *t*_0_ = 0, 5, 10, 15 s (cf. **Figure 4**).

Predictions on the validation sets were quantified using a modified version of the Bernoulli log-likelihood in Equation (15). In the setting of time-varying computations based on the STRF, the filtered stimulus may not depend on a fixed filter **k** but on some filter **k**_*t*_ that may change over time (cf. Equation 2). Thus, the time-dependent Bernoulli log-likelihood, log 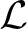
_
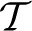
_, is given by

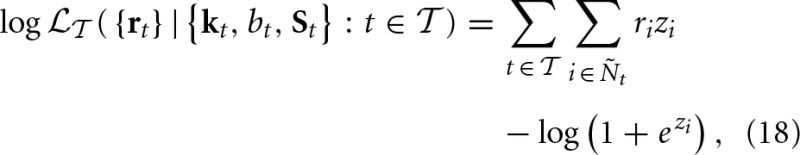



where *z_i_* ≡ **s**_*i*_^T^**k**_*t*_ + *b_t_*, $\tilde{N}$_*t*_ is the test set for each part, and 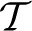 includes all parts. **k**_*t*_ and *b_t_* are local STRF parameters valid for the respective stimulus-response examples. In case the local model **k**_*t*_ provides a better description of time-varying processing than the static model **k**, predictions based on **k**_*t*_ should yield higher predictive power than predictions based on **k**.

#### 2.5.6. Clustering of time-varying STRFs

Local STRFs were clustered using a Gaussian mixture model (GMM) with diagonal covariance matrices. For each data set, all local STRF estimates (the bootstrap estimates derived using a random 90% subset in each part) were pooled and a multivariate mixture of Gaussians, where each Gaussian had the same dimensionality as the STRFs, was fit to the data. The parameters were found using the expectation-maximization (EM) algorithm (Dempster et al., 1977).

We varied the number of multivariate Gaussians from 1 to 20 and used the number that minimized the Bayesian information criterion (BIC). The BIC is defined as

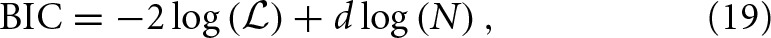



where 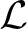 is the likelihood of the data given the model, *d* the number of parameters to be fitted, and *N* the total number of local STRFs (Murphy, 2012). Thus, BIC effectively penalizes overly complex models. The EM algorithm was started 10 times with different initial conditions, and the best solution was used for the analysis. We also tested non-diagonal covariance matrices which did not improve the results. Further, we found that using the Akaike information criterion (AIC) resulted in an artificially high number of clusters with highly correlated cluster centers.

## 3. Results

### 3.1. Simulated data

**Figure 3:**
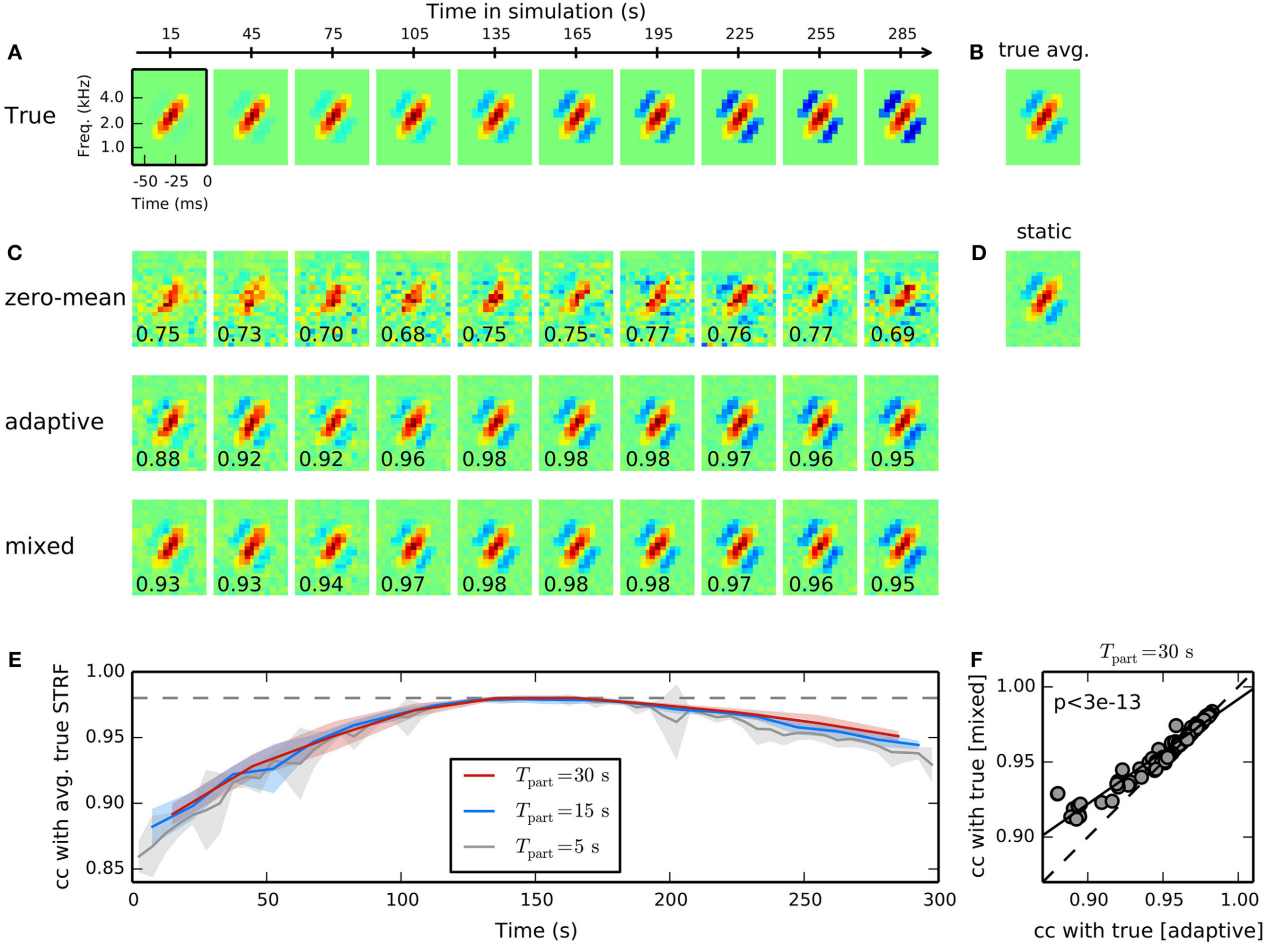
**Time-adaptive STRF estimation from simulated responses to human speech. (A)** Model cell with time-varying STRF whose inhibitory components gradually increase over time. Shown are averaged linear filters for non-overlapping parts of length 30 s. Responses were generated by filtering 300 s of speech spectrograms with the time-varying STRF and applying a static non-linearity to the filtered stimuli. **(B)** The temporal average of the ground-truth time-varying STRF. **(C)** STRF estimates for the different parts of data obtained using a GLM with zero-mean (top), adaptive (middle), and mixed (bottom) prior. For the adaptive and the mixed prior, the static STRF in **(D)** has been used during inference of STRF parameters. The GLM with zero-mean prior produces highly unreliable STRF estimates for the different parts. Both adaptive and mixed prior GLMs allow robust tracking of the time-varying linear filter. Numbers in the lower left corner of each filter estimate indicate correlation between estimated local STRF and mean STRF for each part. The GLM with mixed prior performs marginally better in some cases with strong deviation of the time-varying from the static STRF. **(D)** The static STRF estimated from the whole stimulus-response sequence. **(E)** Dependence of mixed prior performance on part length. Shown are mean and standard deviation of correlations between estimated local and average true STRF across 10 trials. As part length decreases, local STRF estimates become more unreliable as indicated by the noticeable increase in standard deviation. Part lengths of 15 s and longer allowed robust tracking of time-varying processing. **(F)** Comparison of performance of a GLM with adaptive and mixed prior for different human speech stimulus ensembles and different non-linearities. Across all conditions (*N* = 100), the GLM with mixed prior reveals a better reconstruction of the true time-varying STRF (Wilcoxon rank test).

The performance of different priors in the Bernoulli GLM is demonstrated with model simulations. We used a linear-nonlinear model cell with Gabor function-shaped linear STRF and introduced temporal variability by increasing the strength of inhibitory filter components over time (cf. Figure 3A), akin to, e.g., successive stimulus selectivity sharpening (Sadagopan and Wang, 2010). As an example for natural stimuli, we used 300 s of human speech from the TIMIT corpus (Garofolo et al., 1993). Speech utterances were transformed into the time-frequency domain using a gammatone filter bank with logarithmic frequency resolution between 500 Hz and 8000 Hz. The magnitude of the filter bank outputs was logarithmically compressed and subsampled to 250 Hz. After filtering speech spectrograms with the chosen time-varying linear STRF, a non-linear response was obtained by applying a static sigmoid function to the filtered stimuli. Spikes were generated by an inhomogeneous Poisson process on the transformed output.

Figure 3B shows the ground-truth time-varying STRF averaged across time and Figure 3C the static STRF estimate obtained from the entire stimulus-response sequence using a GLM with zero-mean Gaussian prior. The estimated static STRF closely resembles the mean true STRF yielding a correlation of 0.98. Local STRF estimates were obtained by subdividing the whole sequence into 10 non-overlapping parts, each of length 30 s, and estimating STRFs using zero-mean, adaptive, and mixed priors, respectively. The static estimate (cf. Figure 3D) has been used as a prior during inference of GLM parameter with the adaptive and mixed prior approaches.

Figure 3C shows the temporal evolution of the linear filter estimate as obtained with a Bernoulli GLM with zero-mean, adaptive, and mixed priors. STRF estimates produced by the GLM with zero-mean prior tend to be very noisy due to the small number of samples and the highly non-Gaussian structure of speech. In contrast, both adaptive and mixed prior allowed a robust tracking of the true time-varying STRF for the different parts. Correlation between estimated STRF and mean true STRF for each part (cf. Equation 17) verify that the GLM with mixed prior performs slightly better than the GLM with adaptive prior. Note that STRF estimates based on adaptive and mixed prior are biased toward the static STRF. Thus, the proposed approach produces conservative estimates and may underestimate changes in the STRF in some situations.

Reliability of time-varying STRF estimates and, thus, our ability to track them, is determined jointly by variability of the underlying neuronal system and the part length of estimation windows used. To assess the impact of the latter on tracking the simulated system, we repeated the above experiment with part lengths between 5 s and 30 s and evaluated mean correlation with ground truth STRF and its standard deviation; Figure 3E summarizes results obtained with lengths 5 s, 15 s, 30 s. As Expected, standard deviation of STRF estimates tends to increase toward smaller part lengths, with a large increase observed near 10 s length. The results suggest that the proposed method allows time-varying characterization of the investigated system with time-constants as small as about 10 s.

The time-varying STRF estimates revealed by the GLM with adaptive prior (middle row in Figure 3C) and with mixed prior (bottom row in Figure 3C) yield similar correlation values with the true underlying time-varying STRF. However, the GLM with mixed prior reveals a slightly better performance for parts in which the true STRF tends to be more sparse. A summary comparing performance of adaptive and mixed prior GLMs for different sets of human speech stimuli and different non-linearities is shown in Figure 3F. Across all conditions (*N* = 100), the GLM with mixed prior reveals systematically higher correlation values with the true time-varying STRF (*p* < 3 · 10^−13^; paired Wilcoxon test). Thus, the additional hyperparameter in the mixed prior allows more flexible estimation of time-varying STRFs. The benefit of the mixed prior, however, is rather small and on huge data sets the adaptive prior, which has only one hyperparameter, may provide a good estimate of time-varying processing.

### 3.2. Experimental data

**Figure 4:**
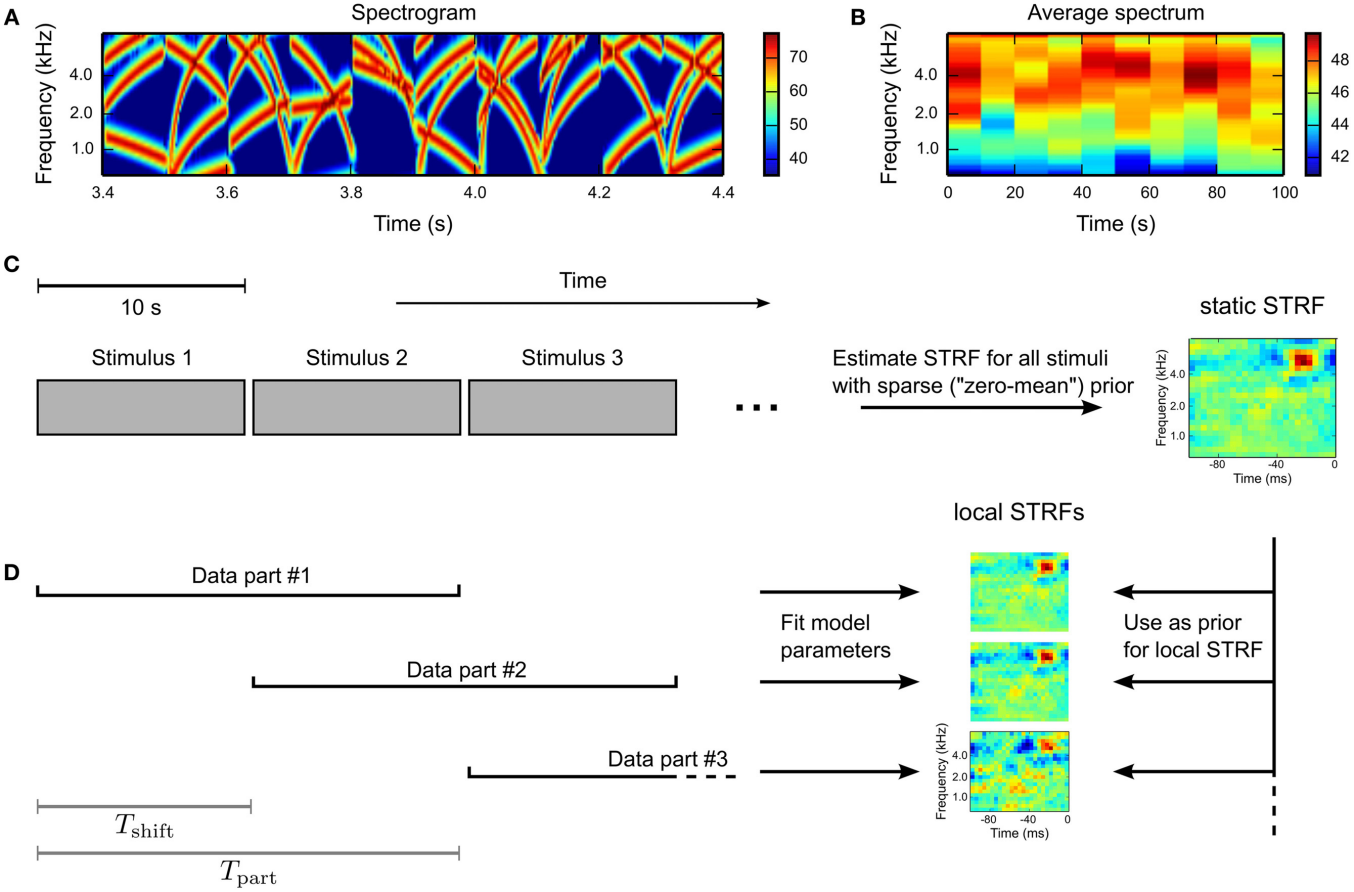
**Experimental design**. Single units in the inferior colliculus (IC) and the primary auditory cortex (A1) in anesthetized Mongolian gerbils were probed with a dynamically fluctuating stimulus ensemble. Acoustic stimuli were composed of random frequency-modulated (FM) tone complexes arranged in 100 ms blocks. The stimulus sequence was interleaved every 10 s by a period of silence (100–1000 ms). **(A)** The spectrogram for a 1 s example. Amplitude values are given in decibel. **(B)** Average spectra for 10 s stimuli indicating stationary characteristics on medium and long time scales. Note the smaller range of amplitude values compared to **(A)**. **(C)** For every unit, an STRF was estimated using the whole stimulus sequence and a GLM with zero-mean prior. Silence periods were not included in the analysis. **(D)** A time-varying STRF was constructed by subdividing the whole stimulus sequence in overlapping parts of length *T*_part_ and temporal shift *T*_shift_. For every part, a local STRF was estimated using a GLM with mixed prior (using the static STRF), yielding a sequence of time-varying STRFs.

To investigate time-varying processing in auditory brain areas, we probed single units in the inferior colliculus (IC) and the primary auditory cortex (A1) in anesthetized Mongolian gerbils with a dynamic, broadband stimulus sequence, whose spectral extent encompassed the frequency response of each unit. The sequence was composed of consecutive 100 ms blocks of frequency-modulated (FM) tone complexes with randomly chosen starting and ending frequencies (for details see Materials and Methods). The spectrogram for a 1 s example segment is shown in Figure 4A. Every 10 s the sequence was interleaved with a period of silence (length between 100 ms and 1000 ms). Average spectra for 10 s stimuli (Figure 4B) demonstrate the fairly stationary long-term characteristic of the stimulus ensemble.

For each unit, a static STRF estimate was obtained using the whole stimulus sequence and a GLM with zero-mean prior (Figure 4C). The stimulus sequence was divided into overlapping parts of length *T*_part_ = 20 s and temporal shift *T*_shift_ = 5 s. Based on the partitioned data, local STRFs were estimated using the Bernoulli GLM with mixed prior (see Equation 16), with the static STRF estimate used as mean in the mixed GLM prior distribution. The principle is illustrated in Figure 4D.

**Figure 5:**
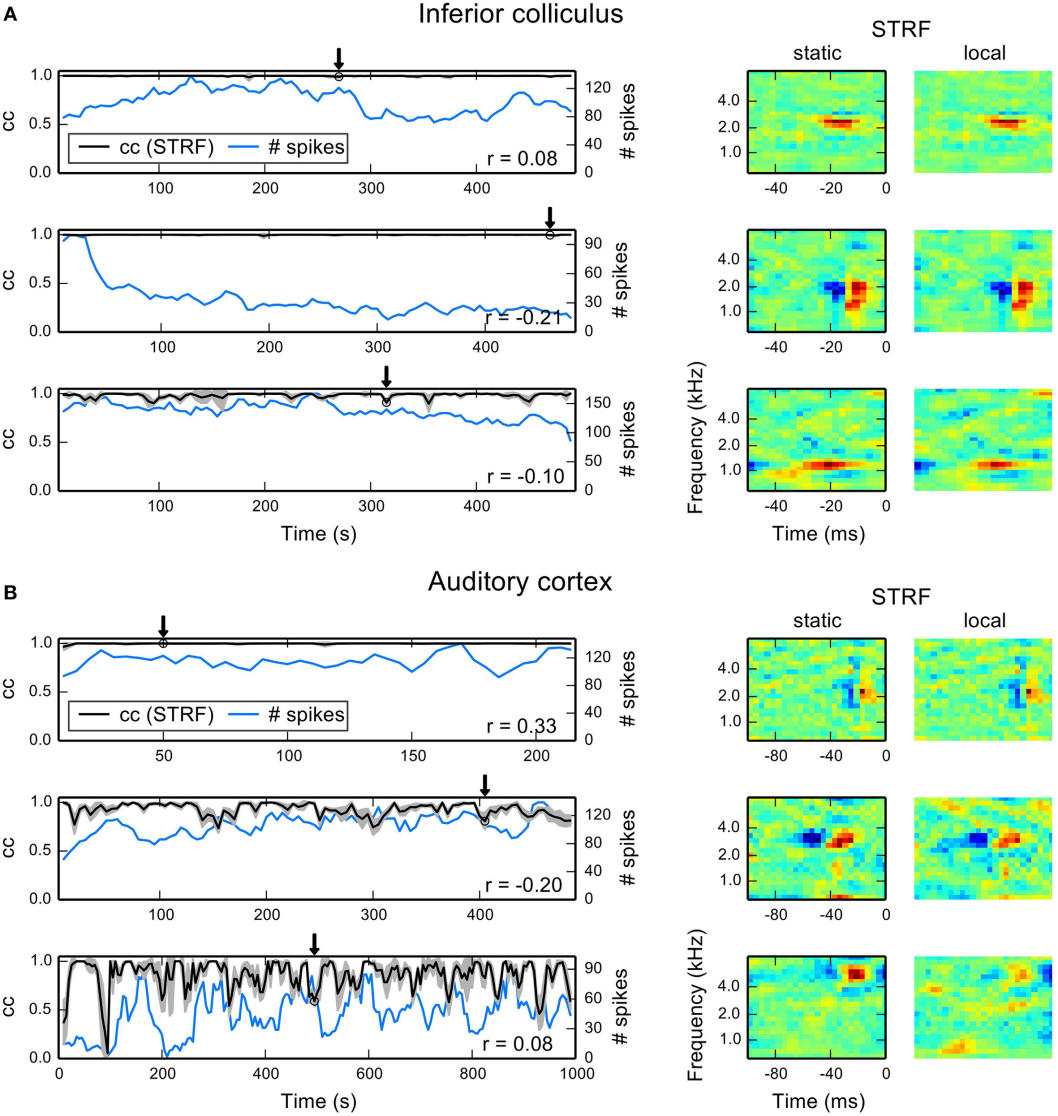
**Examples for time-dependent STRF analysis in auditory midbrain and cortex**. For each part, we repeatedly estimated a local STRF using 90% of the data. To quantify the similarity between local and static STRFs, we used the correlation in terms of normalized subspace projection. Correlation values shown here are mean correlations across all 10 local bootstrap estimates. **(A)** Each row shows time-dependent correlations (denoted by cc) between local STRFs and static STRF (black line) and the corresponding number of spikes in the parts (blue line) for thee IC units. The gray shaded area indicates one standard deviation across the local bootstrap estimates. The arrows indicate the position of the local STRF example shown to the right, together with the static STRF. Numbers in the right lower corners indicate correlations between the cc trace and the number of spikes. **(B)** The same analysis for three A1 units. There is a considerable increase in fluctuations compared to IC examples. The local STRF examples primarily reveal changes in excitatory components at best frequency.

To quantify fluctuations of local STRF estimates, we used correlation in terms of normalized subspace projection between static and local STRFs (cf. Equation 17). To obtain approximate confidence intervals we repeatedly estimated each local STRF using a random subset of 90% of the data. The results reported here represent mean and standard deviation across 10 repetitions. Figure 5A shows temporal evolutions of correlation with the static STRF and number of spikes in each part for three example IC units. The first two units reveal virtually no deviation from the static STRF, although the second unit exhibits adaptation as indicated by a decrease in spike count. The third unit is an example of an IC unit with rather strong fluctuations. Even in this case, the correlation does not fall below 0.8. The local STRF examples for the three units reveal hardly any changes in STRF structure (right column of Figure 5A).

Most A1 units generally showed local STRFs with a higher degree of variability over time than IC units, ranging from time-invariant (first example in Figure 5B) to highly fluctuating local STRFs (third example in Figure 5B). We found parts that show a high degree of variability (indicated by a high standard deviation) and parts that reveal only small variations across the bootstrap estimates. The example STRFs essentially revealed changes in the strength of excitatory STRF components whereas inhibitory regions remained approximately constant. Thus, balance between excitation and inhibition may play an important role in time-varying integration of stimulus features in the cortex. However, we did not find a consistent pattern across all units, and a detailed analysis would require devised experiments beyond the scope of this study.

#### 3.2.1. Population analysis

**Figure 6:**
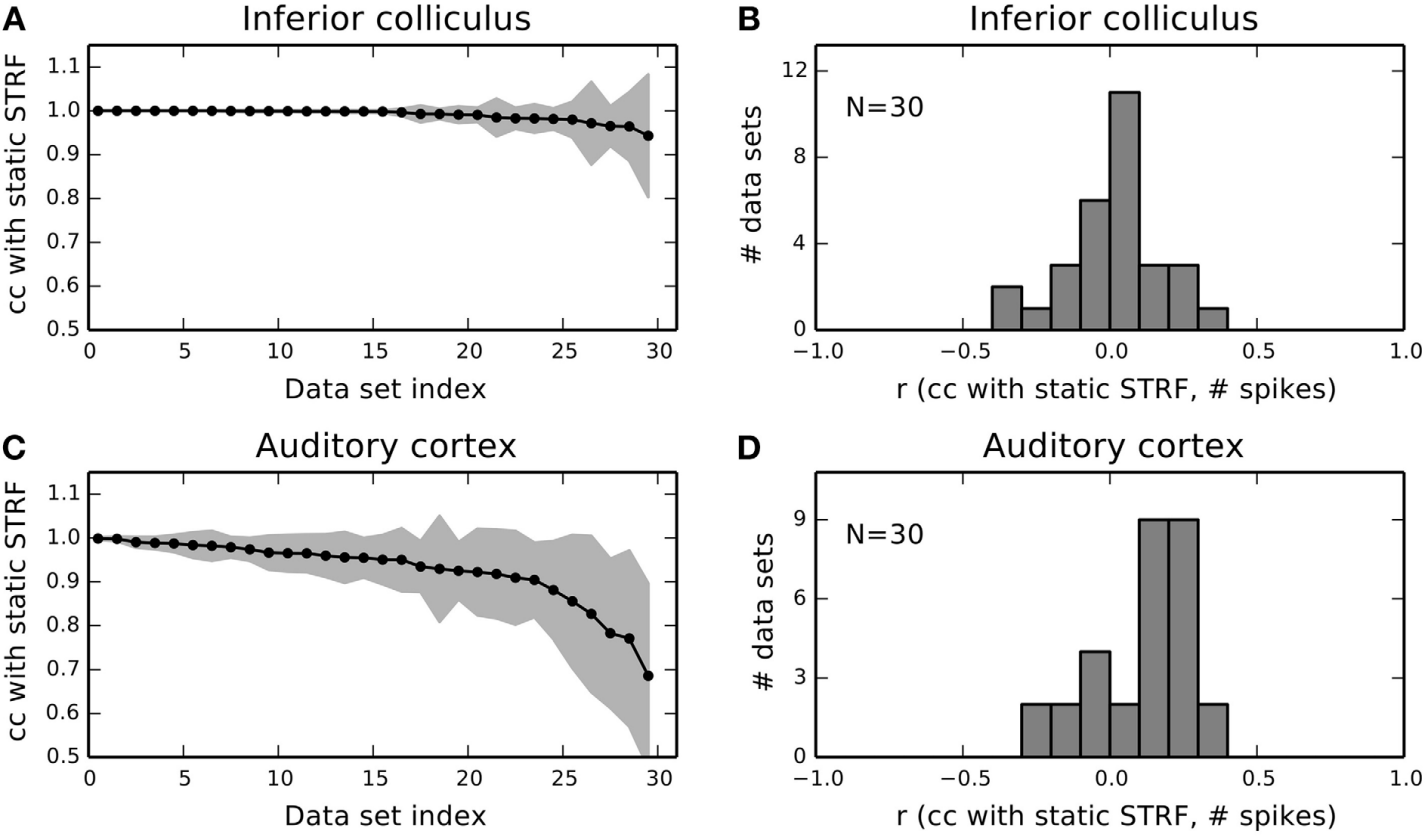
**Population summary for 30 IC and 30 A1 units. (A)** Mean and standard deviation of correlations between local STRFs with the static STRF for each IC data set. Data sets have been sorted by mean correlation. Shaded gray area indicates one standard deviation. **(B)** Distribution of correlations of STRF fluctuations and the number of spikes in each part for 30 IC units. The distribution is approximately Gaussian centered at zero. **(C,D)** The same analysis as in **(A,B)** but for 30 A1 units. A1 units reveal a higher degree of variability compared to IC.

Figure 6A summarizes mean correlation of time-varying with static STRF estimates and corresponding standard deviation for 30 IC units. For the majority of units, mean correlation was not noticeably different from one never below 0.95. In A1, mean correlations and standard deviations revealed much stronger fluctuations across the neural subpopulation (Figure 6C). A noticeable number of units yielded a mean correlation lower than 0.9 and exhibited a considerably larger standard deviation than in IC. We found that only 2% (4%) of the local STRFs pooled across all IC parts (*N* = 2835) have a correlation smaller than 0.9 (0.95) with the static STRF. In A1, about 20% (34%) of the local STRFs for all parts (*N* = 2768) have a correlation of less than 0.9 (0.95). For each part, we used the average correlation across the 10 bootstrapped local STRF estimates.

The variability of A1 responses in terms of the STRF gives rise to the question whether the observed fluctuations may also be explained by the number of spikes. Thus, we calculated the correlation coefficient (denoted by *r* in Figure 5) between time-evolving correlation of local and static STRF and the number of spikes in each part. Figures 6B,D show the distribution of correlation coefficients for IC and A1 units, respectively. In both regions, variability in the STRF is largely uncorrelated with the number of spikes as indicated by the Gaussian-like distributions concentrated around zero (IC) or close to zero (A1). Consequently, a change in spike count does not imply a change in feature integration in terms of the STRF (and vice versa).

#### 3.2.2. The time-varying STRF estimate yields higher predictive power than the static STRF

To verify that the time-varying STRF model provides a meaningful characterization of time-dependent neural processing, we compared prediction performance of local and static STRFs. If changes in STRF structure were exclusively induced by sensory stimuli, we could fit the model on a specific stimulus sequence and validate its predictive power on a repeated sequence. However, as we assume significant modulation of the response by top-down and random network processes (and potentially also other influences, e.g., the employed ketamine anesthesia), we need to adopt a different approach to quantify model performance.

Here we used a cross-validation scheme similar to repeated random subsampling (see Materials and Methods). Briefly, we split the whole stimulus-response sequence into a training (90%) and a validation (10%) set and compared predictions based on the static STRF (estimated using all training data) with predictions based on local STRF estimates (estimated for non-overlapping parts). The static STRF estimate has been used as a prior for local STRF estimates. Thus, the validation set has not been used for estimation of STRF parameters. We repeated the procedure for different randomly selected training/validation sets and different part shifts to cover all samples and all local STRFs.

The Bernoulli log-likelihood (BLL, Equation 15) is a natural measure to evaluate predictions on binary data. Thus, we adapted the BLL to the time-varying scenario (cf. Equation 18). To normalize the log-likelihood for each unit, we subtracted the log-likelihood calculated for predictions on the validation set based on the full STRF estimated using all data (including the validation set). Thus, a value greater than zero indicates that cross-validated local STRF estimates have higher predictive power than the full model that allows the best possible fit on the data by a time-invariant Bernoulli GLM.

**Figure 7:**
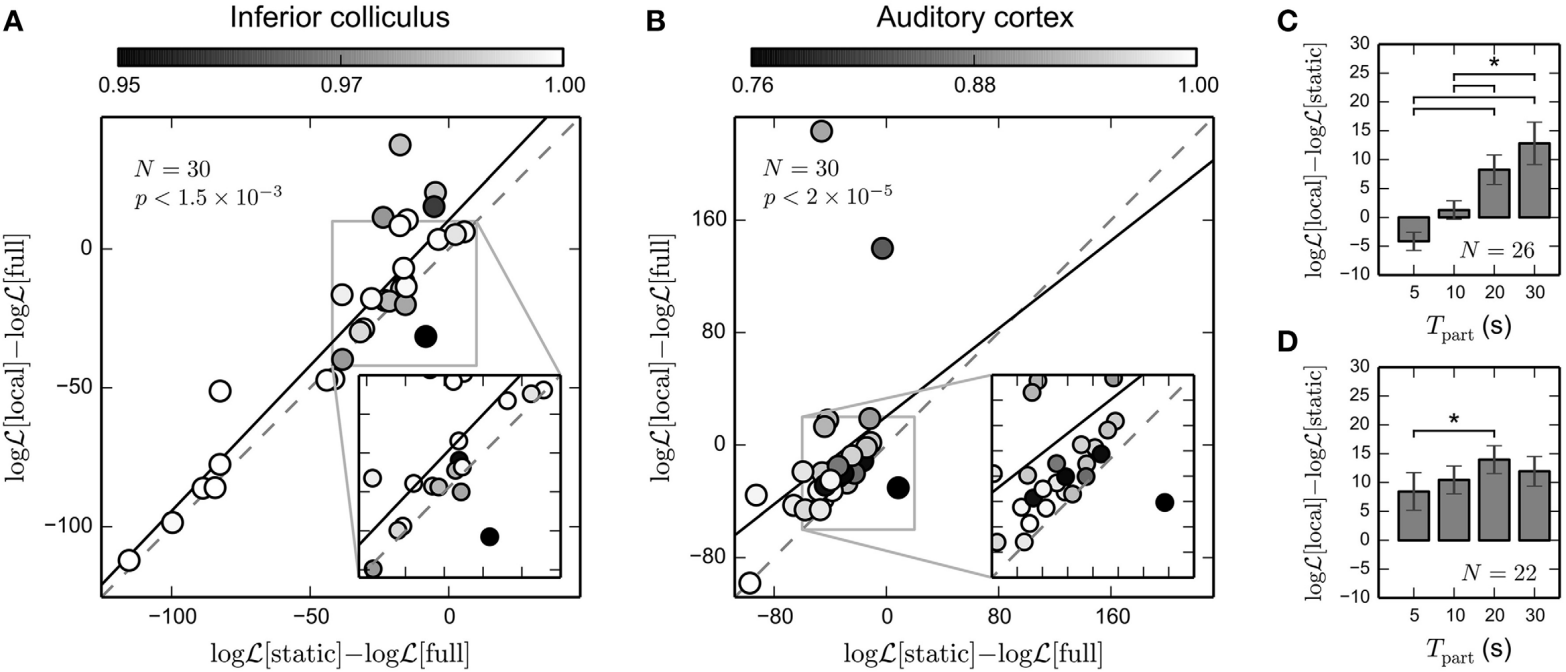
**Predictive power of time-invariant and time-varying STRF model**. We evaluated the predicted Bernoulli log-likelihood (BLL) of local STRFs and static STRF for each unit using repeated random subsampling (see text for details). For each unit, we normalized the log-likelihood by the log-likelihood of the full model learned using all data (including the test data; denoted by “full”). A value greater than zero indicates that even the best static Bernoulli GLM model may not account for potentially time-varying neural processing. **(A)** Normalized BLL for 30 IC units. For the majority of units, predictive power of local STRF estimates is higher than for the static STRF. Across all units, the observed increase in predictive power is significant (Wilcoxon rank test). The black solid line indicates the linear fit. Color encodes mean correlation between local STRFs and static STRF for each unit. Note that due to random sampling, the correlation has been calculated for slightly different subsets than in Figure 6. **(B)** Normalized BLL for 30 A1 units. Compared to IC, there is a considerable increase in predictive power of local STRFs (Wilcoxon rank test). For some units, predicted normalized BLL is greater than zero, implying that a static model is not sufficient to explain the data. **(C)** Average increase in cross-validated log-likelihood of the time-varying over the static model as a function of the interval used to derive the local STRFs for IC data. Only units that contained at least five spikes in each interval were used in the analysis (here: *N* = 26). **(D)** The same as in **(C)** but for the 22 A1 units that contained at least five spikes in each time interval. The time-varying model provided a better description of cortical recordings than the static model, even on small time scales of 5–10 s. ^*^ indicates statistical significance (Wilcoxon rank test; α = 0.05).

Figure 7A shows cross-validated normalized BLL for IC recordings. For the majority of units (26 out of 30), local STRF estimates revealed higher predictive power than the static STRF. In particular, there is a number of units exhibiting relatively high STRF variability (a mean correlation of local STRFs with the static STRF of about 0.95) that showed a high increase in prediction performance. Note that correlation values refer to correlations between local and static STRFs on the training data. Across all units the increase in predictive power is significant (*p* < 1.5 × 10^−3^; paired Wilcoxon test). Similar results were obtained for the 30 A1 units shown in Figure 7B. 28 out of 30 units yielded higher cross-validated normalized BLL for local STRFs than for the static STRF method. The increase in predictive power is highly significant (*p* < 2 × 10^−5^; paired Wilcoxon test). Thus, our results indicate that time-varying STRF estimates based on the GLM with mixed prior provide a robust characterization of time-varying neural integration mechanisms, in particular for cortical data.

To test how prediction performance depends on the part length used to derive local STRF estimates, we repeated the above analysis for different part lengths *T*_part_ = 5 s, 10 s, 20 s, 30 s (cf. Figure 4). The results are summarized in Figures 7C,D for IC and A1 recordings, respectively. In IC, we found a considerable increase in prediction performance of the time-varying model compared to the static model for part lengths greater or equal to 20 s. In the cortex, however, the increase in prediction performance is also evident for smaller part lengths. These findings are consistent with the results obtained using simulated data (cf. Figure 3E). This suggests that the proposed approach is able to capture time-varying processes in the cortex with a characteristic temporal resolution of 5–30 s.

#### 3.2.3. Characterization of fluctuations in time-varying STRFs

The increase in predictive power for local STRFs suggests that changes in STRF structure are correlated with changes in response properties. However, this does not quantify whether or not these variations are systematic, e.g., the local STRF may fluctuate randomly or between distinct states. To quantify changes in local STRFs, we clustered local bootstrap estimates for each unit using a Gaussian mixture model (GMM; see Materials and Methods). If local STRFs were fluctuating between distinct states, we should be able to identify a small number of GMM clusters, and these clusters should repeatedly occur over time. However, in case fluctuations were not governed by such processes, the number of clusters should be rather large, and clusters should not occur more than once.

**Figure 8:**
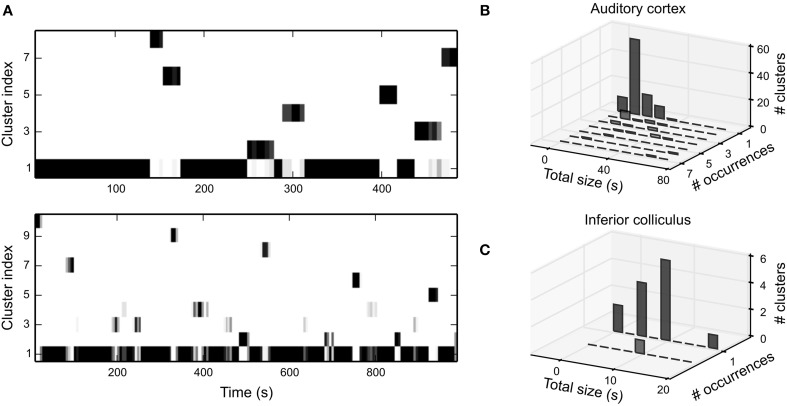
**Clustering of time-varying STRFs. (A)** Temporal evolution of clusters of bootstrapped local STRFs for the latter two A1 example units in Figure 5B. The majority of clusters appeared only once during the whole stimulus sequence. The temporal extent of the clusters indicates that consecutive local STRFs describe very similar states. On a global scale, however, fluctuations appear at random and are not systematic. Clusters have been sorted by total size in descending order. Color encodes the number of bootstrap STRF estimates in the different clusters per time step (white: 0, black: 10). **(B)** Summary of cluster statistics across all clusters for 30 A1 units. Each histogram relates the number of occurrence of clusters to the total temporal extent of the clusters. For each unit, the total time the time-varying STRF was in a specific state (cluster) was estimated by counting all time steps in which more than half of the bootstrap estimates were assigned to the corresponding cluster. Note that we did not include clusters with the static STRF into the analysis (cluster 1 in both examples in **A**). **(C)** Same analysis as in b for 30 IC units. In IC, only a few clusters were identifiable, and virtually all clusters did not occur more than once per stimulus sequence. Thus, in both regions a time-varying STRF analysis does not reveal repeated patterns or long-term changes in STRF structure.

Figure 8A shows the temporal evolution of local STRF clusters for the two latter cortical example units in Figure 5B. In general, we were able to identify distinct clusters that almost never occur multiple times. The temporal extent of many clusters is about the length of the time window of local data. This is likely a result of correlations induced by overlapping parts of the data used to derive local STRFs. However, in some units the temporal extent was clearly larger, and we also find clusters that occurred multiple times (Figure 8B). In IC, we were able to identify only a very small number of clusters, and the extent of the clusters was much smaller than in A1 (Figure 8C). Thus, in both regions we did not find evidence for gradual changes in the STRF or distinct repetitive neural states.

## 4. Discussion

### 4.1. Summary of findings

The purpose of this study was to analyze time-varying neural processing in terms of the STRF. Therefore, we developed a probabilistic approach that characterizes time-varying computations by time-dependent deviations from the standard static STRF. The formulation of time-varying STRF inference in terms of a prior allowed us to apply the proposed approach to a range of models, including linear models and the GLM. Using simulated responses to natural stimuli, we demonstrated that a Bernoulli GLM with mixed prior, a prior that can either be adaptive or zero-mean, provides a means to analyze time-dependent computations, even in case the stimulus ensemble has strong second- and even higher-order correlations across stimulus dimensions. Hence, the proposed approach allows the investigation of time-varying cortical responses to behaviorally relevant stimuli, e.g., con-specific vocalizations or human speech, on medium time scales (10–30 s).

We applied the concept of the time-varying STRF to recordings from IC and A1 in anesthetized Mongolian gerbils. A1 neurons showed a noticeably higher degree of fluctuations in the STRF compared to neurons in IC. In both regions, fluctuations could not be explained by the number of spikes, suggesting that spiking probability is only partially determined by linear feature integration. To test this, we also analyzed how other parameters in the Bernoulli GLM, namely sigmoid non-linearity and spiking threshold, change in situations in which the linear time-varying STRF was highly correlated with the static STRF (data not shown). We found that a change in spike count was mostly correlated with a shift in threshold. In a small subset of units, however, spiking was also affected by a scaling of the non-linearity. Note that the STRF describes effective neuronal processing and its parameters may not directly correspond to a specific neural implementation. Nevertheless, by investigating how the parameters of the linear-nonlinear model change over time, as we have done for the linear part, we may obtain insight into the effective mechanisms underlying these changes.

The time-varying STRF model provided significantly better prediction of IC and A1 neuron responses compared to the time-invariant STRF model. The proposed approach provides a simple and powerful way of extending existing response models for auditory neurons, thus capturing time-dependent coding of these neurons. For a subset of units in both IC and A1, we found that predictions on the validation set based on the time-varying model were better than predictions by a time-invariant model that has also been trained on the validation set. Consequently, for these units a static model seems to be insufficient to account for the neural response. This finding underlines both validity of the proposed approach and the importance of time-varying models to studying neural processing.

A cluster analysis of time-variant STRFs revealed that fluctuations are consistent, i.e., local STRFs estimated in consecutive, overlapping time frames were assigned to the same cluster. Note that the employed GMM did not use temporal information during clustering. As a result, any temporally localized clusters were exclusively induced by structures in the data. These clusters, however, did not show a repeating pattern. In most cases, clusters did not occur multiple times. Consequently, fluctuations were not completely random but there is no evidence for systematic or gradual changes on longer time scales. We would like to emphasize that the employed stimulus ensemble is stationary on longer time scales (cf. Figure 4B) and that experimental design did not induce behavioral relevance to any stimulus part. In case the stimulus ensemble includes relevant context information and strongly varies over time, we would expect both gradual or systematic changes and further evidence of time-dependence in the STRF.

### 4.2. Possible origins of fluctuations in the STRF

The origins of the observed fluctuations may be manifold, and thus we can only speculate. In both IC and A1, neurons were probed using the same stimulus ensemble, and experimental conditions and protocol were identical. Consequently, the observed increase in fluctuations in cortical compared to midbrain neurons is likely a result of characteristic differences between both regions.

Information-theoretic STRF analyses suggest that single cortical neurons may encode multiple features corresponding to multiple STRFs (Atencio et al., 2008, 2012). A possible question in relation to the above findings is whether neurons encode these features simultaneously or whether they “switch” between features at different time instants. We identified distinct local STRF clusters, but these clusters did not frequently occur over time. In most cases, clusters occurred only once. This suggests that the observed fluctuations represent a single fluctuating feature rather than an additional acoustic feature.

Recent studies provide evidence that A1 neurons may respond to features that are weakly correlated with the spectro-temporal amplitude pattern (Chechik et al., 2006; Chechik and Nelken, 2012). For the employed fluctuating stimulus ensemble, such correlations would presumably vary across time as observed for cortical neurons. Another potential source is an increase in context sensitivity through adaptive bottom-up processing. The amplitude of the FM tones was identical throughout the whole stimulus sequence, but the local spectro-temporal contrast varied on a time scale up to seconds (cf. Figure 4A). Such changes may already be sufficient to evoke a change in response properties (Rabinowitz et al., 2011, 2012). Although changes in spectro-temporal contrast have been shown to be mainly compensated for by the neural non-linearity, there may be an interaction with other time-varying processes. Further, the fixed block structure of the stimulus ensemble may evoke adaptation to stimulus statistics in terms of regularity (Ulanovsky et al., 2003, 2004), and this type of adaptation has been found to be more pronounced in the cortex than in IC (Malmierca et al., 2009). The static STRF, a simplified model that primarily assumes time-invariant bottom-up processing, may not be an adequate description of neural processing in such situations.

Response variabilities across neurons are strongly influenced by network fluctuations that operate over a range of spatial and temporal scales, extending in some cases across cortical areas (Zohary et al., 1994; Smith and Kohn, 2008). They may arise from stochastic internal sources, while complex deterministic processes have been suggested to provide a major contribution (Beck et al., 2012; Toups et al., 2012) that may be discernible from random noise (Balaguer-Ballester et al., 2014). These fluctuations are sensitive to global or local internal states (for a review, see Harris and Thiele, 2011), e.g., they are more pronounced and may also change during anesthesia (Ecker et al., 2014) or in response to behavioral salience induced by experimental design (Abolafia et al., 2013). To test whether such effects may contribute to STRF feature integration, we applied the proposed approach to recordings from a multichannel shaft electrode for FM tone complex stimuli in two preparations (data not shown; for a description of the electrode see Happel et al., 2010). If fluctuations in STRFs resulted from global states, there should be non-zero correlations between adjacent electrode channels. We found small correlations of fluctuations between the different channels in the range −0.05 to 0.34. Zohary et al. (1994) observed comparable correlations in sensory cortex for spike count data on smaller time scales (approximately 1–2 s). A theoretical analysis demonstrated that even small correlations may have a distinct impact on global brain performance. Conversely, the observed fluctuations may be a result of global phenomena, e.g., top-down modulation, the employed ketamine anesthesia, or variability in local microcircuits as demonstrated in visual areas (Hansen et al., 2012). The fact that noise correlations are virtually absent in IC (Garcia-Lazaro et al., 2013) supports this hypothesis.

### 4.3. Limitations, extensions, and possible applications

In general, the proposed approach may be applied to any scenario in which sensory feature integration in neurons varies over time under the above constraints. Throughout different stimulus ensembles and different non-linearities, we found 10 s to be a robust lower limit for the proposed approach in the auditory system. Additional parameters, such as data dimensionality, strength of correlations between different stimulus dimensions and the number of spikes in each part, may influence characteristic time scales. In particular, for continuous response signals, e.g., local field potentials within cortex (Arieli et al., 1996) or from the cortical surface (Mesgarani and Chang, 2012), the proposed approach may provide a robust characterization of time-varying processes on smaller time scales than for spiking neurons.

In the context of time-varying neural processing, state-space methods have been of special importance, in particular to study time-varying properties in the hippocampus (Brown et al., 1998, 2001). Due to their recurrent nature, state-space approaches do not require a division of the data into discrete time intervals (Stanley, 2002; Eden et al., 2004), and it is also possible to embed Markovian dynamics into the GLM (Paninski et al., 2010). However, finding solutions the resulting problem usually involves approximations to the posterior and, for small data sets, these may be numerically unstable (Paninski et al., 2010). The proposed approach does not involve any approximations and is very straightforward to implement using standard gradient descent techniques. Further, the formulation of the parameter learning problem in terms of a prior allows to use the approach in a large range of models, including the GLM, but also non-probabilistic approaches, e.g., (Meyer et al., 2014). Thus, in case time-varying processing appears on time scales of about 10 s, the proposed approach represents an alternative to state-space models, in particular for highly non-Gaussian stimulus ensembles that require careful regularization of RF parameters.

The adaptive prior assumes that changes in the STRF are rather small. This is a realistic assumption, e.g., coarse shape and best frequency of a neuron usually remain preserved, even under highly diverse conditions (Escabi et al., 2003; Fritz et al., 2003; Lesica and Grothe, 2008a,b; Rabinowitz et al., 2011; Schumacher et al., 2011). However, when deviations from the static STRF are strong, the adaptive prior will assume the unregularized ML solution. The mixed prior also includes a regularization term enforcing sparseness, thus allowing for more robust estimates in such situations. There are more sophisticated forms of priors, e.g., Laplace prior (Gerwinn et al., 2010; Calabrese et al., 2011), smoothness priors (Sahani and Linden, 2002; Machens et al., 2004), or priors enforcing highly localized RFs (Park and Pillow, 2011), that could be used to increase robustness of local STRF estimates at the expense of more elaborate and computationally expensive optimization schemes.

The hyper parameters of the Bernoulli GLM for both static and local STRF models were found using cross-validation. The parts of the data from which local STRF parameters have been estimated were rather small and even for two hyper parameters this could be done efficiently. However, a computationally efficient alternative to finding hyper parameters of a probabilistic model is Bayesian inference (see Murphy, 2012 for an overview). If neural processing can be described by a linear model, approaches based on empirical Bayes estimation may allow to find multiple hyper parameters more efficiently (Park and Pillow, 2011). In Gerwinn et al. (2010), the posterior distribution over the model parameters of a Poisson GLM has been approximated by a Gaussian using the Expectation Propagation algorithm. It has been demonstrated that the mean of the posterior distribution may have advantages over the MAP estimate in some situations, in particular for the Laplace prior. Bayesian inference also enables the calculation of Bayesian confidence intervals that characterize the uncertainty about the optimal solution. Future research may include Bayesian inference of time-varying RF parameters.

Simultaneous recordings from several layers in A1 revealed that spectro-temporal separability, temporal precision, and feature selectivity varied with layer (Atencio et al., 2009). The proposed approach may provide complementary time-dependent information about coding strategies in different cortical layers. In particular, STRF stationarity may be a layer-dependent property as found in the visual cortex (Hansen et al., 2012). Furthermore, time-dependent analysis of multiple simultaneously recorded neurons in terms of sensory feature integration could help to understand cortical abstraction from spectro-temporal features to auditory identities (Nelken et al., 1999, 2003; Chechik and Nelken, 2012), and temporal dynamics underlying selective attention (Ding and Simon, 2012; Mesgarani and Chang, 2012).

## Author contributions

Conceived and designed the experiments: Arne F. Meyer; Jan-Philipp Diepenbrock; Frank W. Ohl; Jörn Anemüller. Collected the data: Jan-Philipp Diepenbrock. Developed and implemented the methods: Arne F. Meyer. Analyzed the data: Arne F. Meyer. Wrote the paper: Arne F. Meyer. Revised the paper: Arne F. Meyer; Jan-Philipp Diepenbrock; Frank W. Ohl; Jörn Anemüller.

### Conflict of interest statement

The authors declare that the research was conducted in the absence of any commercial or financial relationships that could be construed as a potential conflict of interest.
