# Supplementary material for: Temporal variability of spectro-temporal receptive fields in the anesthetized auditory cortex
Source: Front Comput Neurosci. 2014 Dec 23;8:165. doi: 10.3389/fncom.2014.00165 (PMC4274980; doi:10.3389/fncom.2014.00165)
Supplement: Supplementary file 1 [file DataSheet1.PDF]

Supplementary Material: Temporal variability of spectro-temporal receptive fields in the anesthetized auditory cortex

Arne F. Meyer [1,*], Jan-Philipp Diepenbrock [2], Frank W. Ohl [2,3] and Jörn Anemüller [1]

[1] *Medizinische Physik and Cluster of Excellence Hearing4all, Department of Medical Physics and Acoustics, Carl von Ossietzky University, Oldenburg, Germany*
[2] *Department of Systems Physiology of Learning, Leibniz Institute for Neurobiology, Magdeburg, Germany*
[3] *Department of Neuroprosthetics, Institute of Biology, Otto-von-Guericke University, Magdeburg, Germany*

Correspondence*:
Arne F. Meyer
Carl von Ossietzky University, Department of Medical Physics and Acoustics, Medizinische Physik and Cluster of Excellence Hearing4all, Carl-von-Ossietzky Straße 9-11, D-26129 Oldenburg, Germany, arne.f.meyer@uni-oldenburg.de

SUPPLEMENTAL DATA

DERIVATION OF THE MIXED PRIOR SOLUTION UNDER THE LINEAR-GAUSSIAN MODEL

The mixed prior uses a product of two multivariate Gaussian distributions. For d dimensions and isotropic variance the Gaussian distribution can be written as

$$\mathcal{N}(\mathbf{x}|\mu, \sigma^2\mathbb{I}) = \frac{1}{\sigma^d\sqrt{(2\pi)^d}} \exp\left\{-\frac{1}{2\sigma^2}(\mathbf{x}-\mu)^{\mathrm{T}}(\mathbf{x}-\mu)\right\} \tag{1}$$

where $\mu = (\mu_1, \mu_2, ..., \mu_d)^{\mathrm{T}}$ is a vector of mean values, σ^2 the variance, and $\mathbb{I}$ the identity matrix. The product of the zero-mean prior distribution, $p_\alpha(\mathbf{k}_t|\sigma_\alpha) = \mathcal{N}(\mathbf{k}|\mathbf{0}, \sigma_\alpha^2\mathbb{I})$, and the adaptive prior distribution, $p_\beta(\mathbf{k}_t|\sigma_\beta) = \mathcal{N}(\mathbf{k}|\sigma_\beta^2\mathbb{I})$, is also Gaussian distributed with mean

$$\mu_{\text{mixed}} = \frac{\mathbf{k}\,\sigma_\alpha^2}{\sigma_\alpha^2 + \sigma_\beta^2} \tag{2}$$

and variance

$$\sigma^2_{\text{mixed}} = \frac{\sigma_\alpha^2\,\sigma_\beta^2}{\sigma_\alpha^2 + \sigma_\beta^2}. \tag{3}$$

This can be shown by forming the product of the two distributions and completing the square in the exponent. We do not need the normalization constant because the regularization parameters depend on the

(unknown) noise variance of the data (see Eq. (5) in the main text). Thus, the mixed prior distribution is given by

$$p(\mathbf{k}_t|\mathbf{k}, \sigma_\alpha, \sigma_\beta) \propto \mathcal{N}\left(\mathbf{k}_t| \frac{1}{\frac{\sigma_\beta^2}{\sigma_\alpha^2}+1}\mathbf{k}, \frac{\sigma_\beta^2}{\frac{\sigma_\beta^2}{\sigma_\alpha^2}+1}\mathbb{I}\right) \tag{4}$$

with MAP estimate

$$\hat{\mathbf{k}}_t|\mathbf{k}, \sigma_\alpha, \sigma_\beta = \left(\mathbf{S}^\mathrm{T}\mathbf{S} + c(\frac{\sigma^2}{\sigma_\alpha^2} + \frac{\sigma^2}{\sigma_\beta^2})\mathbb{I}\right)^{-1} \left(\mathbf{S}^\mathrm{T}\mathbf{r} + c\frac{\frac{\sigma^2}{\sigma_\alpha^2} + \frac{\sigma^2}{\sigma_\beta^2}}{\frac{\sigma_\beta^2}{\sigma_\alpha^2}+1}\mathbf{k}\right) \tag{5}$$

where the constant c is the normalization factor of the mixed prior distribution. By defining $\lambda_\alpha = c\frac{\sigma^2}{\sigma_\alpha^2}$ and $\lambda_\beta = c\frac{\sigma^2}{\sigma_\beta^2}$ the above equation can be simplified to Eq. (11) in the main text. Note that for clarity we omitted the time index.
